# Physician awareness of, interest in, and current use of artificial intelligence large language model-based virtual assistants

**DOI:** 10.1371/journal.pone.0320749

**Published:** 2025-05-28

**Authors:** Rachel L. Solmonovich, Insaf Kouba, Ji Y. Lee, Kristen Demertzis, Matthew J. Blitz

**Affiliations:** 1 Northwell, New Hyde Park, New York, United States of America; 2 Department of Obstetrics and Gynecology, South Shore University Hospital, Bay Shore, New York; Zucker School of Medicine at Hofstra/Northwell, Hempstead, NY, United States of America; 3 Biostatistics Unit, Office of Academic Affairs, Northwell Health, New Hyde Park, New York, United States of America; 4 Institute of Health Systems Science, Feinstein Institutes for Medical Research, Northwell Health, New Hyde Park, New York, United States of America; University Hospital of Padova, ITALY

## Abstract

There is increasing medical interest and research regarding the potential of large language model-based virtual assistants in healthcare. It is important to understand physicians’ interest in implementing these tools into clinical practice, so preceding education could be implemented to ensure appropriate and ethical use. We aimed to assess physician 1) awareness of, 2) interest in, and 3) current use of large language model-based virtual assistants for clinical practice and professional development and determine the specific applications of interest and use. Additionally, we wanted to determine associations with age, gender, and role. We conducted a cross-sectional study between 11/08-12/2023 via an anonymous web-based survey that was disseminated among physicians at a large NY healthcare network using snowball sampling. Descriptive and basic inferential statistics were performed. There were 562 respondents, largely males (55.7%), attending physicians (68.5%), and from nonsurgical specialties (67.4%). Most were aware of large language model chatbots (89.7%) and expressed interest (97.2%). Only a minority incorporated it into their practice (21%). Highest levels of interest were for journal review, patient education, and documentation/dictation (88.1-89.5%). The most frequently employed uses were medical information and education and study/research design. Females showed higher interest than males (99.2% vs. 95.5%, p = 0.011). Attendings were more aware of large language models (92.2% vs. 84.2%, p = 0.004), while trainees had increased rates of use (28.8% vs. 17.4%, p = 0.002). Use varied across age brackets, highest among 20-30 year olds (29.1% vs. 13.5%-23.4%, p = 0.018), except for documentation/dictation, where highest use was among the 41-50 year old group (10.5% vs. 2.6%-8.7%, p = 0.047). We concluded that physicians are interested in large language model-based virtual assistants, a minority are implementing it into their practice, and gender-, role-, and age-based disparities exist. As physicians continue to integrate large language models into their patient care and professional development, there is opportunity for research, education, and guidance to ensure an inclusive, responsible, and safe adoption.

## Introduction

ChatGPT (generative pre-trained transformer), an open-access artificial intelligence (AI) deep learning large language model (LLM) released in November 2022, inspired the science community to explore the healthcare potential of LLMs. A Pubmed search for “ChatGPT” yielded more than 3,000 results as of May 2024, with a large amount of data examining the performance of LLMs in passing medical exams or assisting with diagnostics. A number of publications demonstrate how ChatGPT has been found to pass various medical licensing exams, [1–4] suggesting a potential use for medical education and assistance in consultation, diagnosis, and other aspects of patient care. However, there’s a paucity of data related to physicians’ interest in and current use of these tools.

Theoretical advantages for LLMs in the medical field include improved scientific writing, [5] documentation, [1,6,7] dataset analysis, drafting papers, language review, and personalized learning, [5] offering the potential to enhance medical education, clinical decision-making, research, and patient care. [1,8,9] However, concerns exist regarding the implementation of LLMs into clinical practice include ethical, copyright, transparency, and legal issues, and the risk of bias, plagiarism, misinformation, and incorrect citation. [5,10–12] These concerns emphasize the need for oversight, regulations, and boundaries to ensure ethical and transparent usage, as well as quality, relevant, and appropriate results that complement current practice.

Physicians may be unaware that these tools exist as possible aids for their clinical and educational needs, and they may have interest in incorporating them into their practice. Proper education on appropriate use, including awareness of intrinsic biases and limitations and appropriate prompt engineering, should precede incorporation of LLMs into daily practice. Some may already be taking advantage of these services, without fully understanding their potential promise and pitfalls. Understanding physician awareness of, interest in, and current use of LLMs would allow for tailoring implementation strategies and the preceding education necessary for safe, ethical, and appropriate use.

The few studies that have looked at healthcare interest in clinical AI were either limited to trainees, were not specific to physicians, or had a small number of survey respondents. [13–16] Given the theoretical advantages for LLMs in the medical field and the limited data on how practicing physicians perceive and utilize these tools in real-world settings, we aimed to assess United States physicians’ awareness of, interest in, and current use of LLM-based virtual assistants. Secondary objectives were to determine interest and current use of specific education, research, and patient care purposes, and to stratify interest in and current use of LLMs by gender (male versus female), age (by decade), and role (attending physician versus trainee).

## Materials and methods

This was a cross-sectional survey study distributed between November 8, 2023 and December 5, 2023 via institutional email to physicians employed at a large, academic, New York healthcare system. All physicians were eligible to participate, including attendings, fellows, and residents. The Northwell Health institutional review board approved this study and waived the requirement for informed consent.

An established, validated instrument did not exist to address our objectives, so the 15-question survey (S1 File) was developed by the primary author. ChatGPT was utilized during the initial phase of drafting the survey. The final version used in the study was significantly modified to contain questions relevant to our study objectives that were appropriate for our intended survey recipients in a format allowing for ease of completion and data analysis. The survey was reviewed for clarity and comprehensiveness by multiple board-certified attendings, to ensure that the questions presented would satisfy the research objectives. The authors determined that given the exploratory nature and descriptive goals for the study, this was sufficient to finalize the survey design prior to distribution.

The survey link was disseminated through snowball sampling. The survey was first sent out to institutional email addresses of graduate medical education (GME) and department leadership, requesting that they share it with their colleagues. The survey was closed to further responses after 4 weeks.

Before survey initiation, all participants read a short introduction explaining the survey’s purpose and that participation was voluntary and anonymous. The first set of questions covered demographics, such as role, years in practice, specialty, main healthcare setting, age, and gender. Additional questions addressed knowledge of, interest in, and use of LLM-based virtual assistants for various educational and clinical purposes. Levels of interest were ascertained via a 3-point Likert scale (not interested, somewhat interested, very interested). Study data were collected and managed using Research Electronic Data Capture (REDCap) hosted at Northwell Health. [17, 18]

The primary outcomes were rates of physician 1) awareness of, 2) interest in, and 3) current use of LLM-based chatbots. To assess physician interest as a binary variable, a composite variable was created – “no” when answered”not interested” and “yes” when answered “somewhat interested” or “very interested” for any of the questions addressing interest. The secondary outcomes were 1) interest levels in and 2) current use of specific educational and clinical applications. Sub-analyses were performed to determine whether results were associated with gender, age, and role. Age groups were analyzed by decades for practicality and ease of interpretation and comparison.

**Fig 1:**
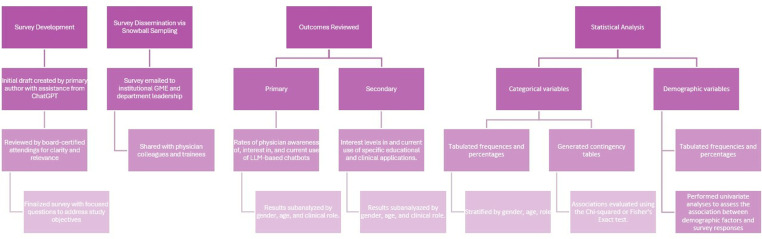
Study Methodology.

For categorical variables, frequencies and percentages were tabulated for the overall population and stratified by gender, age, and role. Additionally, frequencies and percentages of demographic variables were tabulated for respondents who reported current LLM use. A total of 51 univariate analyses were performed to assess the association between demographic factors (gender, age, and role) and the survey responses. For each pair of categorical variables, contingency tables were generated, and associations were evaluated using the Chi-squared test. When the expected counts in any cell were less than 5, Fisher’s Exact test was used instead. All analyses were conducted using SAS V9.4 (SAS Institute Inc., Cary NC.) A p-value < 0.05 was considered statistically significant. The study methodology is delineated in Fig 1.

## Results

A total of 562 physicians completed the survey. The majority of respondents self-identified as male (55.7%), attending physician role (68.5%), and nonsurgical specialty (67.4%). Baseline demographics are described in Table 1.

**Table 1:** Baseline demographics of survey responders (n = 562).

Characteristic	No. (%)
Role	
Attending physician	385 (68.5)
<1 year out of training	12 (3.1)
1-5 years out of training	58 (15.1)
6-10 years out of training	81 (21.0)
11-20 years out of training	94 (24.4)
20^+^ years out of training	140 (36.4)
Trainee	177 (31.5)
Fellow	42 (23.7)
Resident	135 (76.3)
Specialty^a^	
Surgical^b^	182 (32.6)
Nonsurgical	376 (67.4)
Main healthcare setting^c^	
Hospital	427 (76.0)
Clinic/outpatient	202 (35.9)
Age, years	
20-30	110 (19.6)
31-40	172 (30.6)
41-50	124 (22.1)
>50	156 (27.8)
Gender^d^	
Male	312 (55.7)
Female	243 (43.4)
Other/prefer not to say	5 (0.9)
Aware of LLM-based virtual assistants	504 (89.7)
Use of LLM-based virtual assistants	118 (21.0)

^a^Frequency missing = 4.

^b^The American College of Surgeons recognizes general surgery, obstetrics and gynecology, neurosurgery, ophthalmology, orthopedics, otolaryngology, plastic surgery, and urology among surgical specialties.

^c^Multiple answer choices may be selected.

^d^Frequency missing = 2.

Physicians from 32 specialties completed the survey. The most represented specialties were obstetrics and gynecology (14.2%), pediatrics (12.0%), general surgery (8.6%), anesthesiology (7.9%), internal medicine (7.4%), and orthopedics (5.2%). A few identified as being from an unlisted specialty (4.1%).

### Primary outcomes

Most were aware of LLM-based virtual assistants for medical support (89.7%, n = 504) and were interested in using such assistance (97.2%, n = 546). Approximately 1/5 of respondents were already incorporating LLM assistants into their routines (21.0%, n = 118).

### Secondary outcomes

#### 1. Interest.

**Fig 2:**
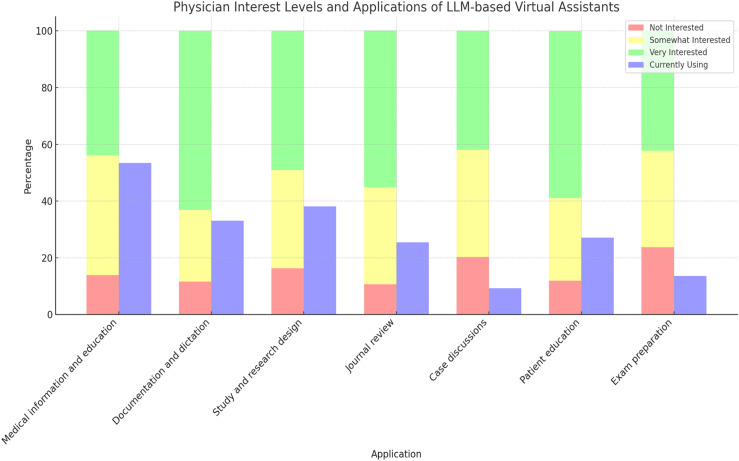
Physician interest levels and applications of LLM-based virtual assistants. Interest was expressed for all listed purposes, with highest levels for journal review, patient education, and documentation/dictation. Among the physicians currently using LLM assistants (n = 118), the most frequently used applications were medical information and education, study/research design, and documentation/dictation. *Interest levels were obtained from the entire study cohort (n = 562). **Current use rates were obtained from a sub-cohort of physicians who endorsed currently using LLM-based virtual assistants, and multiple applications could be endorsed by the same physician (n = 118).

All specialties had high interest rates (85.71%-100%), shown in Table 2, and interest was expressed for all listed purposes. Highest levels of interest were for journal review (89.5%), patient education (88.1%), and documentation/dictation (88.4%). (Table 3, Fig 2)

**Table 2:** Interest and current use by medical specialty.

Specialty	Interest, No. (%)	Use, No. (%)
Anesthesia	40 (90.9)	3 (6.8)
Cardiology	19 (100)	3 (15.8)
Critical care	22 (100)	1 (4.6)
Dermatology	3 (100)	0 (0)
Emergency medicine	22 (100)	8 (36.4)
Endocrinology	2 (100)	1 (50.0)
Family medicine	10 (90.9)	3 (27.3)
Gastroenterology	11 (100)	2 (18.2)
Geriatrics	1 (100)	0 (0)
Hematology/oncology	13 (92.9)	8 (57.1)
Infectious disease	2 (100)	0 (0)
Internal medicine	40 (97.6)	19 (46.3)
Interventional radiology	4 (100)	1 (25.0)
Nephrology	1 (100)	1 (100)
Neurology	12 (85.7)	3 (21.4)
Neurosurgery	5 (100)	3 (60.0)
Obstetrics/gynecology	77 (97.5)	10 (12.7)
Ophthalmology	2 (100)	1 (50.0)
Orthopedics	29 (100)	4 (13.8)
Otolaryngology	14 (100)	1 (7.1)
Palliative care	1 (100)	0 (0)
Pathology	9 (100)	4 (44.4)
Pediatrics	65 (97.0)	10 (14.9)
Physical medicine and rehabilitation	2 (100)	0 (0)
Plastic surgery	3 (100)	1 (33.3)
Psychiatry	26 (100)	9 (34.6)
Pulmonology	16 (100)	4 (25.0)
Radiology	16 (94.1)	6 (35.3)
Rheumatology	3 (100)	1 (33.3)
Sports medicine	1 (100)	0 (0)
Surgery	46 (95.8)	7 (14.6)
Urology	2 (100)	0 (0)
Other	23 (100)	3 (13.0)

**Table 3:** Physician interest in LLM-based virtual assistants (n = 562).

Application	No. (%)		
	Not Interested	Somewhat Interested	Very Interested
Medical information and education	78 (13.9)	237 (42.2)	247 (44.0)
Documentation and dictation^a^	65 (11.6)	142 (25.3)	354 (63.1)
Study and research design^b^	91 (16.3)	194 (34.6)	275 (49.1)
Journal review^c^	59 (10.6)	191 (34.2)	309 (55.3)
Case discussions^a^	114 (20.3)	212 (37.8)	235 (41.9)
Patient education^a^	67 (11.9)	164 (29.2)	330 (58.8)
Exam preparation	133 (23.7)	191 (34.0)	238 (42.4)

^a^Frequency missing = 1.

^b^Frequency missing = 2.

^c^Frequency missing = 3.

Medical information and education, such as to review medical concepts, terminology, procedures, and treatment options.

Documentation and dictation, such as procedure notes, progress notes, and surgical dictation summaries.

Study and research design, such as with literature reviews, IRB applications, research design, and drafting research protocols, abstracts, and manuscripts.

Journal review, to help with summarizing and understanding complex medical studies and research papers.

Case discussions, such as to obtain differential diagnoses, potential treatment options, and to help practice communication and patient counseling skills.

Patient education, such as to obtain patient-friendly explanations for common conditions and procedures.

Exam preparation, such as practice questions and explanations.

#### 2. Current use.

Among the physicians currently using LLM assistants (n = 118), the majority were male (57.8%), attending physicians (56.8%), and from nonsurgical specialties (76.9%). Most use was among the 31-40 years age bracket (30.5%). Among the specialties with the highest response rates, use ranged from 6.82%-46.34%, (anesthesiology and internal medicine, respectively). (Table 2). The most frequently used applications were medical information and education (53.4%), study/research design (38.1%), and documentation/dictation (33.1%). (Table 4)

**Table 4:** Characteristics of physicians currently using LLM-based virtual assistants (n = 118).

**Characteristic**	n (%)						
**Attending physician**, years out of training	<1	1-5 years	6-10 years	11-20 years	20 +		
	1 (1.5)	10 (14.9)	16 (23.9)	20 (29.9)	20 (29.9)		
**Trainee**	**Fellow**	**Resident**					
	12 (10.2)	39 (33.1)					
Specialty^a^	Surgical^b^	Nonsurgical					
	27 (23.1)	90 (76.9)					
**Main healthcare setting** ^c^	**Hospital**	**Clinica/outpatient**					
	93 (78.8)	43 (36.4)					
**Age,** years	**20-30**	**31-40**	**41-50**	**>50**			
	32 (27.1)	36 (30.5)	29 (24.6)	21 (17.8)			
**Gender** ^d^	**Male**	**Female**	**Other/prefer not to say**				
	67 (57.7)	47 (40.5)	2 (1.7)				
**Application** ^c^	**Medical information and education**	**Documentation and dictation**	**Study and research design**	**Journal review**	**Case disucssions**	**Patient education**	**Exam preparation**
	63 (53.4)	39 (33.1)	45 (38.1)	30 (25.4)	11 (9.3)	32 (27.1)	16 (13.6)

^a^Frequency missing =  1.

^b^The American College of Surgeons recognizes general surgery, obstetrics and gynecology, neurosurgery, ophthalmology, orthopedics, otolaryngology, plastic surgery, and urology as surgical specialties.

^c^Multiple answer choices may be selected.

^d^Frequency missing = 2.

Sub-analyses (**Table 5**)

**Table 5:** Inferential statistics by gender, role, and age bracket.

	Gender No. (%)			Role, No. (%)			Years, No. (%)				
	Male (n = 312)	Female (n = 243)	P Value	Attending (n = 385)	Trainee (n = 177)	P Value	20-30 (n = 110)	31-40 (n = 172)	41-50 (n = 124)	>50 (n = 156)	P Value
Aware of LLM-based virtual assistants (yes)	286 (91.7)	212 (87.2)	0.089	355 (92.2)	149 (84.2)	0.004	93 (84.6)	151 (87.8)	114 (91.9)	146 (93.6)	0.07
Interest in LLM-based virtual assistants (yes)	298 (95.5)	241 (99.2)	0.011	371 (96.4)	175 (98.9)	0.097	110 (100)	166 (96.5)	121 (97.6)	149 (95.5)	0.167
Medical information and education			0.688			0.021					0.021
Not	45 (14.4)	33 (13.6)		64 (16.6)	14 (7.9)		5 (4.6)	33 (19.2)	19 (15.3)	21 (13.5)	
Somewhat	126 (40.4)	107 (44.0)		158 (41.0)	79 (44.6)		48 (43.6)	69 (40.1)	58 (46.8)	62 (39.7)	
Very	141 (45.2)	103 (42.4)		163 (42.3)	84 (47.5)		57 (51.8)	70 (40.7)	47 (37.9)	73 (46.8)	
Documentation and dictation			0.903			0.022					0.001
Not	37 (11.9)	27 (11.1)		54 (14.0)	11 (6.3)		8 (7.3)	16 (9.4)	10 (8.1)	31 (19.9)	
Somewhat	76 (24.4)	63 (25.9)		98 (25.5)	44 (25.0)		29 (26.4)	33 (19.3)	34 (27.4)	46 (29.5)	
Very	198 (63.7)	153 (63.0)		233 (60.5)	121 (68.8)		73 (66.4)	122 (71.4)	80 (64.5)	79 (50.6)	
Study and research design			0.011			0.002					0.006
Not	64 (20.6)	27 (11.1)		76 (19.8)	15 (8.5)		8 (7.3)	23 (13.5)	23 (18.7)	37 (23.7)	
Somewhat	100 (32.2)	92 (37.9)		132 (34.4)	62 (35.2)		36 (32.7)	65 (38.0)	38 (30.9)	55 (35.3)	
Very	147 (47.3)	124 (51.0)		176 (45.8)	99 (56.3)		66 (60.0)	83 (48.5)	62 (50.4)	64 (41.0)	
Journal review			0.015			0.002					0.012
Not	43 (13.8)	16 (6.6)		49 (12.8)	10 (5.7)		7 (6.4)	17 (9.9)	14 (11.4)	21 (13.6)	
Somewhat	108 (34.7)	82 (33.7)		140 (36.6)	51 (29.0)		26 (23.6)	60 (35.1)	41 (33.3)	64 (41.3)	
Very	160 (51.5)	145 (59.7)		194 (50.7)	115 (65.3)		77 (70.0)	94 (55.0)	68 (55.3)	70 (45.2)	
Case discussions			0.185			0.772					0.246
Not	67 (21.5)	47 (19.3)		81 (21.1)	33 (18.6)		16 (14.6)	44 (25.6)	22 (17.9)	32 (20.5)	
Somewhat	106 (34.0)	101 (41.6)		145 (37.8)	67 (37.9)		42 (38.2)	56 (32.6)	49 (39.8)	65 (41.7)	
Very	139 (44.6)	95 (39.1)		158 (41.2)	77 (43.6)		52 (47.3)	72 (41.9)	52 (42.3)	59 (37.8)	
Patient education			<0.001			0.464					0.236
Not	53 (17.0)	14 (5.8)		50 (13.0)	17 (9.6)		7 (6.4)	25 (14.5)	12 (9.7)	23 (14.8)	
Somewhat	81 (26.1)	81 (33.3)		113 (29.4)	51 (28.8)		30 (27.3)	47 (27.3)	38 (30.7)	49 (31.6)	
Very	177 (56.9)	148 (60.9)		221 (57.6)	109 (61.6)		73 (66.4)	100 (58.1)	74 (59.7)	83 (53.6)	
Exam preparation			<0.001			<0.001					0.007
Not	100 (32.1)	33 (13.6)		110 (28.6)	23 (13.0)		16 (14.6)	38 (22.1)	28 (22.6)	51 (32.7)	
Somewhat	96 (30.8)	93 (38.3)		128 (33.3)	63 (35.6)		33 (30.0)	62 (36.1)	43 (34.7)	53 (34.0)	
Very	116 (37.2)	117 (48.2)		147 (38.2)	91 (51.4)		61 (55.5)	72 (41.9)	53 (42.7)	52 (33.3)	
Use of LLM-based virtual assistants	67 (21.5)	47 (19.3)	0.537	67 (17.4)	51 (28.8)	0.002	32 (29.1)	36 (20.9)	29 (23.4)	21 (13.5)	0.018
Medical information and education	40 (12.8)	20 (8.2)	0.084	40 (10.4)	23 (13.0)	0.363	14 (12.7)	18 (10.5)	17 (13.7)	14 (9.0)	0.591
Documentation and dictation	22 (7.1)	16 (6.6)	0.829	24 (6.2)	15 (8.5)	0.332	7 (6.4)	15 (8.7)	13 (10.5)	4 (2.6)	0.047
Study and research design	30 (9.6)	15 (6.2)	0.141	20 (5.2)	25 (14.1)	<0.001	17 (15.5)	15 (8.7)	10 (8.1)	3 (1.9)	0.001
Journal review	17 (5.5)	12 (4.9)	0.789	12 (3.1)	18 (10.2)	<0.001	12 (10.9)	10 (5.8)	7 (5.7)	1 (0.6)	0.003
Case discussions	8 (2.6)	3 (1.2)	0.265	6 (1.6)	5 (2.8)	0.314	4 (3.6)	2 (1.2)	2 (1.6)	3 (1.9)	0.53
Patient education	14 (4.5)	18 (7.4)	0.143	13 (3.4)	19 (10.7)	<0.001	11 (10.0)	13 (7.6)	5 (4.0)	3 (1.9)	0.022
Exam preparation	8 (2.6)	6 (2.5)	0.944	7 (1.8)	9 (5.1)	0.031	7 (6.4)	3 (1.7)	5 (4.0)	1 (0.6)	0.029

#### Gender.

There was no evidence of a statistically significant association between gender and awareness of LLM-based virtual assistants.

**Fig 3:**
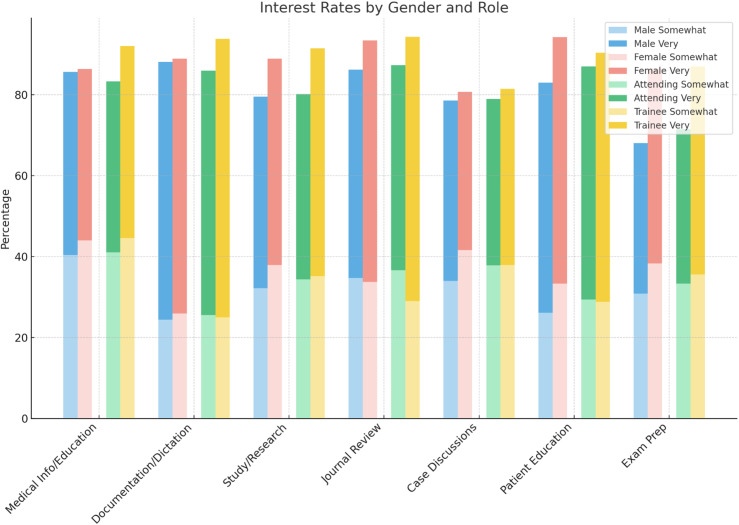
Physician Interest Rates by Gender and Role. Males were less likely to show interest for the following domains: study/research design (p = 0.011), journal review (p = 0.015), patient education (p < 0.001), and exam preparation (p < 0.001). More trainees were very interested for the following uses: medical information and education (p = 0.021), documentation/dictation (p = -0.022), study/research design (p = 0.002), journal review (p = 0.002), and exam preparation (p < 0.001).

Females were more interested than males (99.2% vs. 95.5%, p = 0.011) in using LLM-based virtual assistants. When evaluating level of interest for specific applications, males were less likely to show interest for the following domains: study/research design (20.6% vs. 11.1%, p = 0.011), journal review (13.8% vs. 6.6%, p = 0.015), patient education (17.0% vs. 5.8%, p < 0.001), and exam preparation (32.1% vs. 13.6%, p < 0.001). (Fig 3) There was no evidence of an association between current use of LLM-based virtual assistants and gender.

#### Role.

There was a statistically significant association between role, attending versus trainee, and awareness of LLM-based virtual assistants (92.2% vs. 84.2%, respectively; p = 0.004).

While overall interest in using LLM-based assistants did not significantly differ by role, significant associations were found when analyzing interest levels for specific purposes. Specifically, more trainees were very interested for the following uses: medical information and education (47.5% vs 42.3%, p = 0.021), documentation/dictation (68.8% vs. 60.5%, p = -0.022), study/research design (56.3% vs. 45.8%, p = 0.002), journal review (65.3% vs. 50.7%, p = 0.002), and exam preparation (51.4% vs. 38.2%, p < 0.001). (Fig 3) There was a statistically significant association between role and current use of LLMs, with fewer attendings using LLM assistants compared to trainees (17.4% vs. 28.8%, p = 0.002). Within the specific applications for usage, there were significant associations revealed for the following domains: study and research design (5.2% vs. 14.1%, p < 0.001), journal review (3.1% vs. 10.2%, p < 0.001), patient education (3.4% vs. 10.7%, p < 0.001), and exam preparation (1.8% vs. 5.1%, p = 0.031).

#### Age.

There was no statistically significant association found between age group and awareness of LLM-based virtual assistants.

All individuals between 20-30 years olds were interested, while those in the other age groups expressed varying rates of interest (95.5%-97.6%, p = 0.167). When evaluating level of interest for specific purposes, there were several statistically significant associations between age group and interest level, including for medical information and education, documentation and dictation, study and research design, journal review, and exam preparation.

There was a statistically significant association between age and current use of LLMs. Among respondents aged 20-30 years, 29.1% currently use LLM-based virtual assistants, while only 20.9% in the 31-40 age group, 23.4% in the 41-50 age group, and 13.5% in the 50 + age group use LLM assistance (p = 0.018). When analyzing the specific domains for use, only medical information and education and case discussions were not significant.

## Discussion

### Principal findings

Physicians from a wide variety of specialties completed the survey. The majority of participants were aware of and interested in LLM-based virtual assistants for clinical and educational purposes, while only a minority were already incorporating it into their practice. Highest interest levels were observed for journal review, patient education, and documentation and dictation. The most common uses were for medical information and education, study and research design, and documentation and dictation. Sub-analyses revealed significant gender-, role-, and age-based differences in awareness, interest, and current use of LLM-based virtual assistants.

The differences observed in the sub-analyses in interest and use of LLM virtual assistants likely reflect a combination of generational attitudes toward technology, professional dynamics, and different demands and workflows. Trainee physicians typically fall within the younger age range, and the findings from the sub-analyses align closely with the interest and usage rates observed in the youngest cohort of physicians, as expected. Younger physicians displayed higher usage rates, possibly due to their increased exposure to technology during training and greater comfort integrating digital tools into their practice. An exception was the higher use of documentation and dictation tools among 41-50 year olds, which might reflect their position as mid-career professionals balancing high clinical loads with administrative tasks, making such tools particularly valuable. This older age group is likely the last to have trained before the implementation of the Health Information Technology for Economic and Clinical Health Act, requiring them to adapt to new electronic medical record systems. As a result, further integration of LLM assistants may have been a natural progression for them. The higher interest levels among female physicians may originate from a greater recognition of the potential for such tools to enhance efficiency in tasks traditionally associated with administrative burden, which often disproportionally impact female physicians. [19]

### Results in the context of what is known

ChatGPT amassed a historic amount of users in a brief period, 1 million users within 5 days and 100 million users in two months. [20] Our study demonstrates both interest and use among physicians across specialties. Studies have shown that LLMs have great potential to support research, because they can explore literature and generate hypotheses, handle complex data and extract useful information, and translate complicated findings into more easily understandable language. [21] In a survey by Banerjee et al., the majority of trainee doctors agreed that AI would have an overall positive impact on their training and education. They were most optimistic that clinical AI would enhance their training by improving the efficiency of research, freeing up time to spend on other educational activities, and keeping up with evidence-based practices. [14] The small survey by Spotnitz concluded that practicing clinicians, roles were not specified, were encouraging of using LLMs, especially in assistive roles, but there is need for human oversight. [16] The most positively rated uses were clinical practice and education tasks. Among our surveyed resident and attending physicians, these were also areas of high interest.

A survey by Temsah et al. of healthcare workers in Saudi Arabia found that most were comfortable incorporating ChatGPT into their practice but expressed concerns about credibility and the sources of provided information. Among their survey respondents, which was inclusive of medical students, nurses, technicians, therapists, pharmacists, and physicians, 18.4% were using ChatGPT, and 84.1% of those who were not using it yet were expecting to in the future. [15] Our results are concordant: physicians are interested in using LLMs for a variety of clinical and educational applications, and a minority of them already do. Chen et al. showed via an international web-based questionnaire that physicians and medical students had a positive but reserved attitude toward the application of clinical AI, but they lacked practical experience. They reported a similar clinical AI utility rate of 20% but a much lower awareness rate (38% vs. 89.7%) than our physicians-only cohort. [13]

### Clinical implications

Physicians express high interest levels in integrating LLMs into their practice, and a minority already have. Implementing and expanding LLM use in the domains that physicians expressed interest in, and concordantly spend the bulk of their time, could be time-saving and free up time for other purposes. It could also enhance the physician-patient relationship by improving patient education, [1,6,7] comprehension, and retention. Our survey respondents expressed a high level of interest in using LLM assistants for patient education, and a recent study showed that LLMs have potential to generate proficient medical counseling templates in Spanish for patients [22] as well as generate useful handoff notes to reduce physician documentation burden. [23] These are examples in which LLMs can be applied clinically and impact patient care.

Possible reasons for the discrepancy between awareness and interest versus current use rates include lack of training, institutional hesitancy, technological limitations, and ethical and accuracy concerns. However, with high interest within the healthcare community, it is prudent that the adoption of LLMs in medicine be shaped by medical professionals who can impart the appropriate training data and testing prior to their integration into medical practice. [24] Tailoring implementation strategies based on distinct preferences and needs within roles and age groups, as well as focusing on identified areas of interest, may help ensure higher uptake with appropriate preceding education to ensure safe and ethical use.

It is important to consider the ethics involved with integration of LLMs into clinical practice. Significant ethical concerns exist, such as the possibility of perpetuating biases related to race, sex, language, and culture given that their responses reflect their training data, which originates from high-income English-speaking countries. This also increases the risk for limited perspectives and inability to generalize responses to people from other regions of the world. [12] Furthermore, with AI technology in general, there are privacy and confidentiality concerns related to sharing patient information and images, as well as economic and accessibility disparities that may widen for those who cannot afford or access these AI tools. [25] The epistemic opacity, which is the inability to understand how algorithms make decisions, and whom to assign responsibility when error occurs are additional ethical concerns, [26] which need to be addressed prior to broad integration into medical practice.

AI is an innovative tool with potential to revolutionize modern healthcare practices by enhancing both provider and patient experiences and outcomes. It should be seen as an adjunct resource, like textbooks and other digital tools, and integrated thoughtfully within the broader spectrum of clinical decision-making aids. Explainable AI, which helps clinicians understand the AI’s decision-making process, can support physicians by fostering informed clinical practice, thereby eliminating the need to choose between reliance on opaque algorithms or avoiding these valuable tools. [27] LIME (Local Interpretable Model-agnostic Explanations) and SHAP (SHapley Additive exPlanations) are widely used techniques for explaining machine learning model predictions. LIME approximates model behavior using simpler surrogate models to generate quick, interpretable insights, shedding light on the factors contributing to a particular prediction made by a given machine learning or deep learning model. [28] SHAP explains the importance of each individual feature in the context of a particular prediction, identifying key factors exerting the most substantial influence on a given prediction to reveal the inner workings of machine learning or deep learning models. [29] Although LLMs do not inherently use SHAP or LIME to generate responses, they can be applied externally to explain the outputs of such models when interpretability is required and to enable greater transparency and trust in their applications. Additionally, given that LLMs interact directly with their users, they can clearly convey the reasoning behind their conclusions. By sharing reference sources, LLMs can further enhance transparency and trust, allowing users to validate their claims.

### Research implications

Our study found that most physicians are aware of and interested in LLM-based chatbots for healthcare purposes, yet only a minority are already using them. The discrepancies observed between demographic groups suggest that the adoption of LLM-based tools may be influenced by generational factors within medicine, in addition to practical needs, which warrants further exploration.

Future research can confirm these findings and investigate the underlying reasons for the discrepancy between the high levels of awareness and interest and their relatively low current use rates. Additionally, studies should explore causal factors influencing LLM adoption and continue to explore the benefits of incorporating LLMs into physician practice, such as saving time and money, which can be allocated to other purposes, as well as physician professional satisfaction. Future projects could also collect qualitative insights about the perspectives of physicians regarding LLM adoption. With increased uptake of LLMs into physician practice, studies exploring the limitations and ethical implications of LLM-based virtual assistants in healthcare should be performed to provide reassurance. Furthermore, additional investigation can provide insight into variations in trends and opinions among medical specialties and geographical locations, as well as among other healthcare providers, such as mid-level providers and nurses.

### Strengths and limitations

Our method of survey dissemination via participant self-selection and snowball sampling, which may promote responses of like-minded individuals, as well as only surveying physicians within a NY healthcare system, may limit the generalizability of our results. Additionally, due to our survey distribution methods, an accurate denominator needed to calculate a response rate could not be determined. However, a strength of our study is the large number of responses from physicians across different practice types, specialties, and with varying levels of experience.

## Conclusions

Our study summarizes the current awareness, interest, and adoption of LLM-based virtual assistants by physicians. Physicians express high interest levels in integrating LLMs into their practice, with a notable gap between the amount of interest and actual use rates, both of which are associated with gender, role, and age variations. There is also discordance between which LLM applications physicians are interested in versus what they are currently using. There is still opportunity to implement the necessary research, education, and guidance to ensure an inclusive, beneficial, responsible, and safe adoption of LLMs into physician education and clinical practice.

## Supporting information

S1 File Survey assessing physician interest in and use of AI-powered virtual assistants. (PDF)
