# Supplementary material for: Physician awareness of, interest in, and current use of artificial intelligence large language model-based virtual assistants
Source: PLoS One. 2025 May 28;20(5):e0320749. doi: 10.1371/journal.pone.0320749 (PMC12118853; doi:10.1371/journal.pone.0320749)
Supplement: S1 File — (PDF) [file pone.0320749.s001.pdf]

Survey Assessing Physician Interest in Utilizing AI-powered Virtual Assistants

1. Role:
    - ☐ Attending physician
        - Years out of training
            - ☐ <1 year
            - ☐ 1-5 years
            - ☐ 6-10 years
            - ☐ 11-20 years
            - ☐ 20+ years
    - ☐ Fellow physician
    - ☐ Resident physician
2. Specialty:
    - ☐ Anesthesiology
    - ☐ Cardiology
    - ☐ Critical care
    - ☐ Dermatology
    - ☐ Emergency medicine
    - ☐ Endocrinology
    - ☐ Family medicine
    - ☐ Gastroenterology
    - ☐ Geriatrics
    - ☐ Hematology Oncology
    - ☐ Infectious disease
    - ☐ Internal medicine
    - ☐ Interventional radiology
    - ☐ Nephrology
    - ☐ Neurology
    - ☐ Neurosurgery
    - ☐ Obstetrics and gynecology
    - ☐ Ophthalmology
    - ☐ Orthopedics
    - ☐ Otolaryngology
    - ☐ Pain medicine
    - ☐ Palliative care
    - ☐ Pathology
    - ☐ Pediatrics
    - ☐ Physical medicine and rehabilitation
    - ☐ Plastic surgery
    - ☐ Psychiatry
    - ☐ Pulmonology
    - ☐ Radiology
    - ☐ Rheumatology
    - ☐ Sports medicine
    - ☐ Surgery
    - ☐ Urology
    - ☐ Other
3. Main healthcare setting

- ☐ Hospital
- ☐ Clinic/outpatient

4. Age
   - ☐ 20-30 years old
   - ☐ 31-40 years old
   - ☐ 41-50 years old
   - ☐ >50 years old
5. Gender
   - ☐ Male
   - ☐ Female
   - ☐ Other
   - ☐ Prefer not to say
6. Have you heard of AI-powered virtual assistants for medical support, like ChatGPT or similar tools?
   - ☐ Yes
   - ☐ No
7. How interested are you in utilizing AI assistance for the following purposes?

	Not Interested	Somewhat Interested	Very Interested
Medical information and education, such as to review medical concepts, terminology, procedures, and treatment options	○	○	○
Documentation and dictation, such as procedure notes, progress notes, and surgical dictation summaries	○	○	○
Study and research design, such as with literature reviews, IRB applications, research design, and drafting research protocols, abstracts, and manuscripts	○	○	○
Journal review, to help with summarizing and understanding complex medical studies and research papers.	○	○	○
Case discussions, such as to obtain differential diagnoses, potential treatment options, and to help practice communication and patient counseling skills.	○	○	○
Patient education, such as to obtain patient-friendly explanations for common conditions and procedures	○	○	○
Exam preparation, such as practice questions and explanations	○	○	○

8. Do you already utilize AI assistance for any of those purposes?
   - ☐ Yes
     - Which?
       - ☐ Medical information and education

    - ☐ Documentation and dictation
    - ☐ Study and research design
    - ☐ Journal review
    - ☐ Case discussions
    - ☐ Patient education
    - ☐ Exam preparation
- ☐ No
